# Foam sclerotherapy compared with liquid sclerotherapy for the treatment of lower extremity varicose veins: A protocol for systematic review and meta analysis

**DOI:** 10.1097/MD.0000000000020332

**Published:** 2020-05-29

**Authors:** Minglei Bi, Danyi Li, Zhenyu Chen, Yanjin Wang, Jizhen Ren, Weina Zhang

**Affiliations:** Department of Plastic Surgery, The Affiliated Hospital of Qingdao University, Qingdao, China.

**Keywords:** lower extremity, meta-analysis, sclerotherapy, varicose veins

## Abstract

**Background:**

There is a continued discussion on which is the best sclerosant to treat lower extremity varicose veins. Therefore, we did this meta-analysis to determine that foam sclerotherapy versus liquid sclerotherapy, which could perform better in the treatment of lower extremity varicose veins.

**Materials and methods:**

We independently searched 5 databases from inception to February 1, 2019, for randomized controlled trials and prospective controlled trials for comparing foam sclerotherapy and liquid sclerotherapy for the treatment of lower extremity varicose veins. The Newcastle-Ottawa Scale (NOS) was used to assess the quality of studies. The primary outcome and secondary outcomes were analyzed using stata 15.0. This meta-analysis was performed according to Cochrane Handbook.

**Results:**

There were significant differences in effective rate (*P* < .001, odd ratios = 5.64, 95% confidence interval = 3.93–8.10) and incidence rate of pain (*P* = .030, odd ratios = 1.52, 95% confidence interval = 1.04–2.21) between foam sclerotherapy and liquid sclerotherapy. And there were no significant differences among local inflammation (*P* = .896, rate difference = 0.00, 95% confidence interval = –0.03 to 0.03), thrombophlebitis (*P* = .90, rate difference = 0.00, 95% confidence interval = −0.02 to 0.02) and hyperpigmentation (*P* = .336, rate difference = 0.05, 95% confidence interval = −0.05 to 0.14).

**Conclusions:**

Although foam sclerotherapy has a higher incidence rate of complications, it could achieve a more stable clinical efficacy in the treatment of lower extremity varicose veins than liquid sclerotherapy.

## 1 Introduction

Lower extremity varicose veins are the condition where blood flow refluxes and distal veins engorge because pliable valves fail to prevent retrograde blood flow. It will lead to pathological changes of abnormal veins such as expansion, denaturation, and twist. Generally, lower extremity varicose veins is a common disease and affects up to 25% women, especially those people who always stand for a long time.^[1]^ Furthermore, it can cause the deterioration of the quality of life and other serve complications such as chronic venous ulceration, haemorrhage, venous thromboembolism and the limitation of activity.^[2]^

The variety of therapeutic strategies have ranged from more invasive surgical methods such as high ligation and stripping, stab avulsion phlebectomy, valvuloplasty and valve transplantation, to less invasive therapies such as radiofrequency ablation (RFA), endovenous laser therapy (EVLA), and compression therapy.^[3,4]^ Although there are many existing referral guidelines, the majority of them are “localism” and not evidence based.^[5]^ Therefore, clinical managements of this kind of diseases are driven by doctors’ experience and patients’ economic capabilities mostly. As a traditional and effective method, venous sclerotherapy, which contained polidocanol (POL) and sodium tetradecyl sulfate (STS), has enjoyed a great popularity among above-mentioned treatments because of its simple procedures and positive clinical effects, it could cause endothelial damage which leads to fibrosis of the vessel.^[6]^

However, since Orbach invented foam sclerotherapy by mixing detergent sclerosants with the usually air,^[7]^ there has been a hot debate on the clinical efficacy for lower extremity varicose veins of foam sclerotherapy versus liquid sclerotherapy.^[8]^ Nevertheless, no authorized data could be found to put an end to this debate because there are few multi-center trials with a large size of patients, lacking sufficient scientific evidence to support. To explore this question, we did this meta-analysis of randomized controlled trials (RCTs) or prospective controlled trials (PCTs) to compare foam sclerotherapy and liquid sclerotherapy, determining which is the optimal method to treat lower extremity varicose veins.

## 2 Materials and methods

### 2.1 Eligibility criteria

Studies were included if they were RCTs or PCTs comparing foam sclerotherapy with liquid sclerotherapy in the treatment of lower extremity varicose veins. We excluded any other types of studies such as review, editorial, case report, observational study et al. Inclusion criteria were:

1. the study comparing the effective rate or incidence rate of complications of foam sclerotherapy and liquid sclerotherapy.

Exclusion criteria were:

1. non-English language articles,
2. only described foam sclerotherapy or liquid sclerotherapy,
3. randomized trials comparing foam sclerotherapy and liquid sclerotherapy in which both effective rate and incidence rate of complications were not evaluated.

### 2.2 Information sources

For this meta-analysis, we searched 5 database including PubMed, MEDLINE, Web of Science, EMBASE, and Cochrane Library from inception to April 1, 2019. Several keywords were used during the search procedure and we had English language restriction. Only RCTs or PCTs that compared foam sclerotherapy with liquid sclerotherapy were searched.

### 2.3 Search strategy

The following concrete search strategy was used in PubMed, which was made on the basis of the requirements of the Preferred Reporting Items for Systematic Reviews and Meta-Analysis (PRISMA) statement.^[9,10]^ ((((“Sclerotherapy”[Mesh]) OR Sclerotherapies)) AND ((“Varicose Veins”[Mesh]) OR (((((Varicose Vein) OR Vein, Varicose) OR Veins, Varicose) OR Varix) OR Varices))) AND Foam.

### 2.4 Study selection

Two reviewers (Bi and Li) independently looked through the titles and abstracts of all citations according to the inclusion and exclusion criteria strictly. Full-text of all possible qualified studies was screened by both authors individually. If there was any divergence, the senior reviewer (Chen) would judge it objectively. Studies which met all the inclusion criteria could be collected for this meta-analysis.

### 2.5 Data extraction process and quality measurement

Data were collected and evaluated respectively by two authors (Zhang and Wang) and disagreements would be solved by the third author (Chen). The following information should be extracted:

1. author's name, year, region, number of participants, sex, study design, follow-up duration;
2. the effective rate and incidence rate of complications such as pain, local inflammation, hyperpigmentation and thrombophlebitis.

If necessary, we would contact with corresponding authors for more available information. Otherwise, Newcastle-Ottawa Scale (NOS) was used to assess the quality of all included studies.^[11]^ This scale contains three aspects: selection (0–4 scores), comparability (0–2 scores), outcomes (0–3 scores). The maximal scores were 9 and studies which were more than 6 scores were considered as high quality articles.

### 2.6 Statistical analysis and additional analysis

This meta-analysis was performed using Stata 15.0. Odd ratios (OR) were used to analyze dichotomous data (effective rate, incidence rate of pain) and rate difference (RD) were used to analyze other dichotomous data (local inflammation, hyperpigmentation, and thrombophlebitis), with 95% confidence interval (CI).^[12]^ Otherwise, *P* value < .05 was considered significant. As for heterogeneity, chi-squared test was applied to evaluate it. If *I*^2^ > 50%, it was considered suggestive of statistical heterogeneity, the random-effect model should be applied. The fixed-effect model should be used if *I*^2^ < 50% because there was no significant statistical heterogeneity.^[13]^ In addition, we evaluated publication bias of this meta-analysis by Egger test.^[14]^ We also performed sensitivity analysis to assess the robustness of results.

## 3 Results

### 3.1 Screening and inclusion of studies

**Figure 1:**
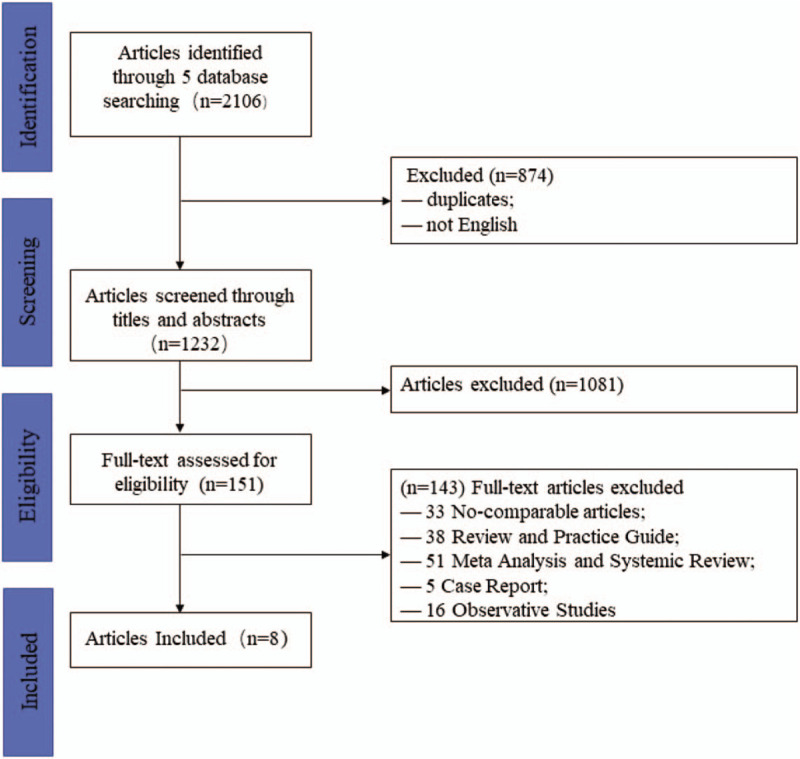
Flow diagram of study selection.

The flow diagram of our literatures searching process was displayed in Figure 1. First, our search had identified 2106 records through 5 database. Secondly, 874 records were excluded because of duplication. Then, after a precise screening through titles and abstracts, there were 151 full-text articles reminded which would be assessed for eligibility. Finally, we had a strict requirement of the type of included studies so such other types of studies as review, letter, observative studies, case report et al would be excluded.

After the careful and strict searching process, 8 studies with 1089 participants (female 80.52%), completed from 2003 to 2018 among 5 different countries (Europe 7, Asia 1), with the mean follow-up period of 10 months, met all the aforementioned inclusion criteria in this meta-analysis. All included articles were full-text studies which were only written in English, and there were 6 RCTs and 2 PCTs. In addition, the mean score of NOS was 7 of 9 and 6 studies were deemed as high quality (>6 scores), the rest were moderate quality. Characteristics of studies included were generally shown in Table 1. Furthermore, we had extracted other important information such as different techniques to produce foam, different diagnosis standards of lower extremity varicose veins among included studies, which were all manifested in Table 2.

**Table 1:**
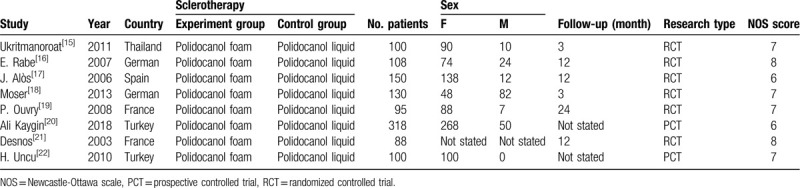
Characteristics of studies included in this meta-analysis.

**Table 2:**
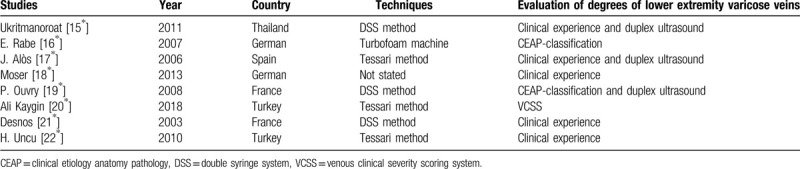
Different techniques to produce foam sclerosant and diagnosis standards of lower extremity varicose veins among included studies.

### 3.2 Primary outcome

#### 3.2.1 Effective rate

**Figure 2:**
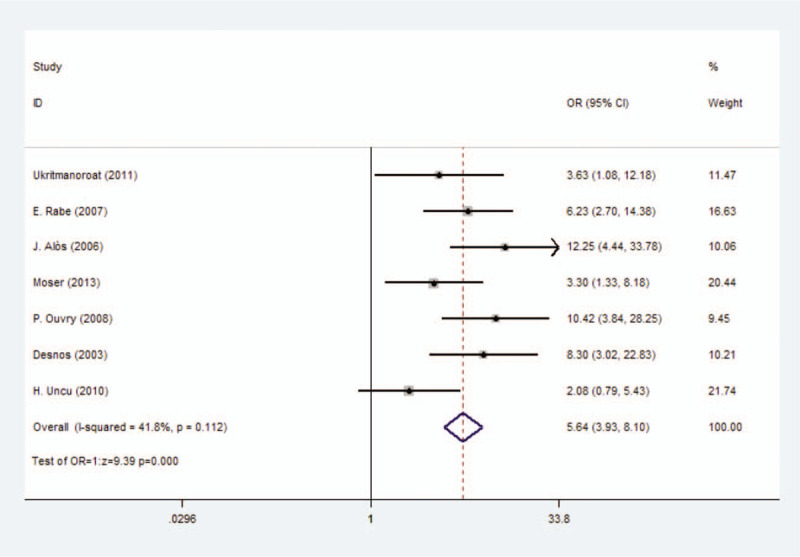
Forest plot to assess effect rate between foam sclerotherapy and liquid sclerotherapy.

The clinical efficacy of foam sclerotherapy or liquid sclerotherapy could be illustrated by this outcome completely and it could be calculated easily by such formula as n(effective events)/n(total events). Seven studies with 771 participants reported this outcome. We used fixed-effect model (*I*^2^ = 41.8%) and there was a significant difference between foam sclerotherapy and liquid sclerotherapy, with OR = 5.64 (*P* < .0001, 95% CI = 3.93–8.10) (Fig. 2) (Table 3).

**Table 3:**
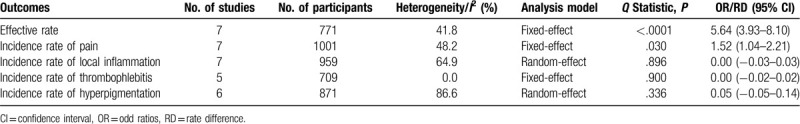
Pooled outcomes indicators for lower extremity varicose veins patients.

### 3.3 Secondary outcomes

#### 3.3.1 Incidence rate of pain

**Figure 3:**
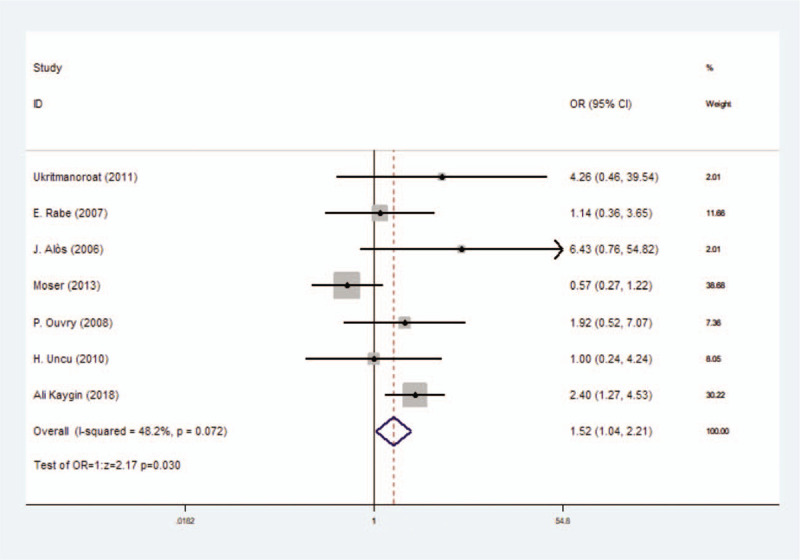
Forest plot to assess incidence rate of pain between foam sclerotherapy and liquid sclerotherapy.

It included 7 studies with 1001 participants and fixed-effect model (*I*^2^ = 48.2%) was used. Results showed that there was a significant difference between foam group and liquid group, with OR = 1.52 (*P* = .030, 95% CI = 1.04–2.21) (Fig. 3) (Table 3).

#### 3.3.2 Incidence rate of local inflammation

**Figure 4:**
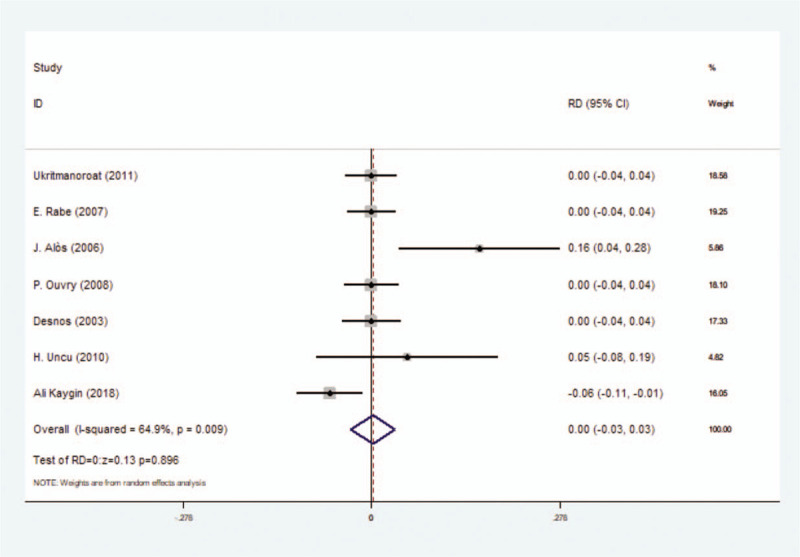
Forest plot to assess Incidence rate of local inflammation between foam sclerotherapy and liquid sclerotherapy.

This outcome contained 7 included studies as well as 959 participants. After meta-analysis on incidence rate of local inflammation outcome, no significant difference was found between foam sclerotherapy and liquid sclerotherapy, with RD = 0.00 (*P* = .896, 95% CI = −0.03 to 0.03) and we used random-effect model (*I*^2^ = 64.9%) (Fig. 4) (Table 3).

#### 3.3.3 Incidence rate of thrombophlebitis

**Figure 5:**
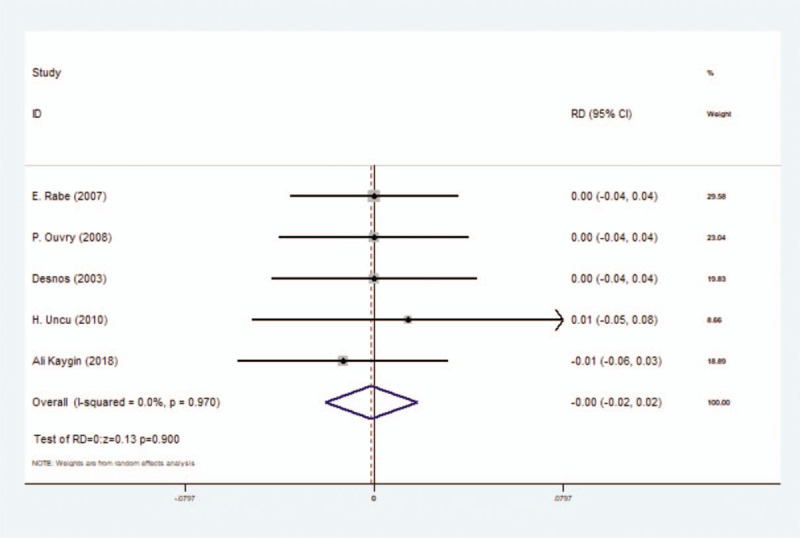
Forest plot to assess Incidence rate of thrombophlebitis between foam sclerotherapy and liquid sclerotherapy.

Five studies assessed thrombophlebitis after sclerotherapy of lower extremity varicose veins, including 709 participants totally. When we compared foam sclerotherapy with liquid sclerotherapy, we chose fixed-effect model because of its low heterogeneity (*I*^2^ = 0.0%) and there was no significant difference between foam group and liquid group, with RD = 0.00 (*P* = .900, 95% CI = −0.02 to 0.02) (Fig. 5) (Table 3).

#### 3.3.4 Incidence rate of hyperpigmentation

**Figure 6:**
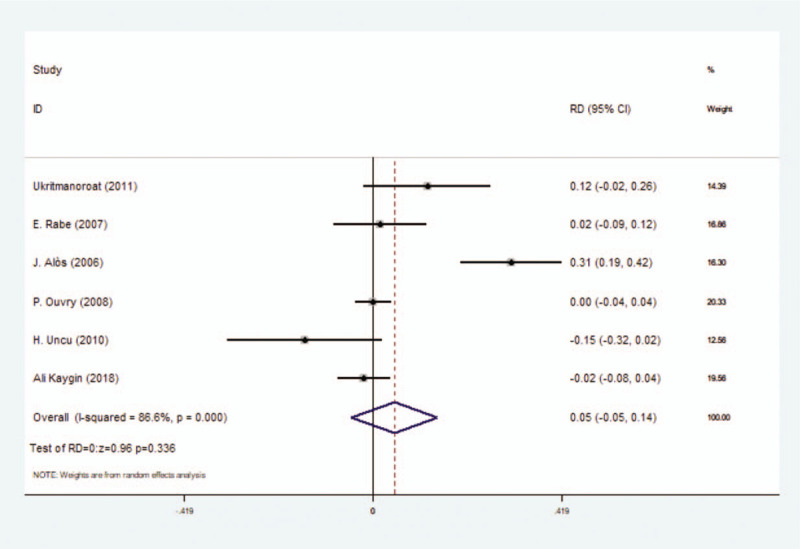
Forest plot to assess Incidence rate of hyperpigmentation between foam sclerotherapy and liquid sclerotherapy.

This secondary outcome could be extracted from 6 included studies with 871 participants in this meta-analysis which existed in the comparison between foam group and liquid group, using the random-effect model because of its high heterogeneity (*I*^2^ = 86.6%). However, there was no significant difference between these two groups, with RD = 0.05 (*P* = .336, 95% CI = −0.05 to 0.14) (Fig. 6) (Table 3).

### 3.4 Publication bias and sensitivity analysis

**Figure 7:**
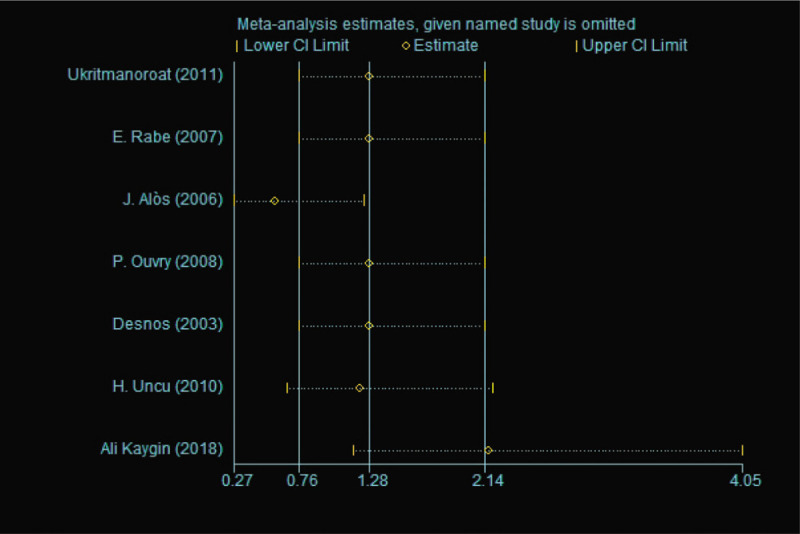
Sensitivity analysis of Incidence rate of local inflammation between foam sclerotherapy and liquid sclerotherapy.

**Figure 8:**
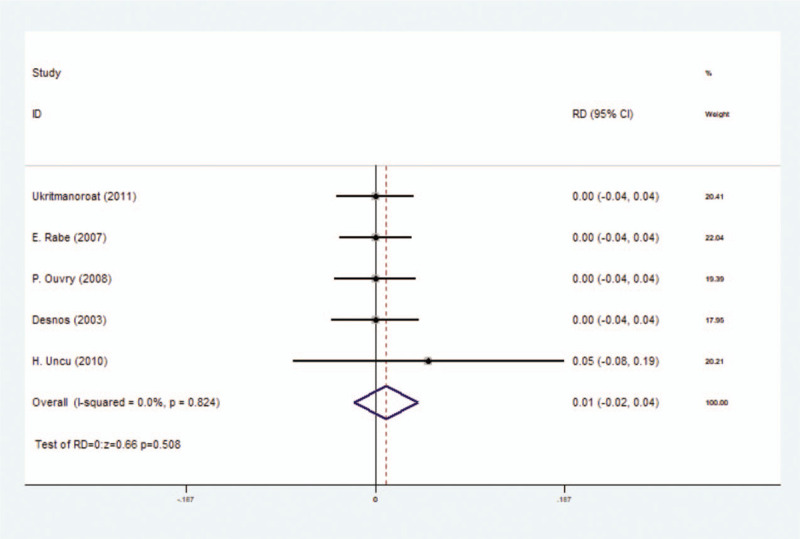
Forest plot to assess Incidence rate of local inflammation between foam sclerotherapy and liquid sclerotherapy after exclusion.

**Figure 9:**
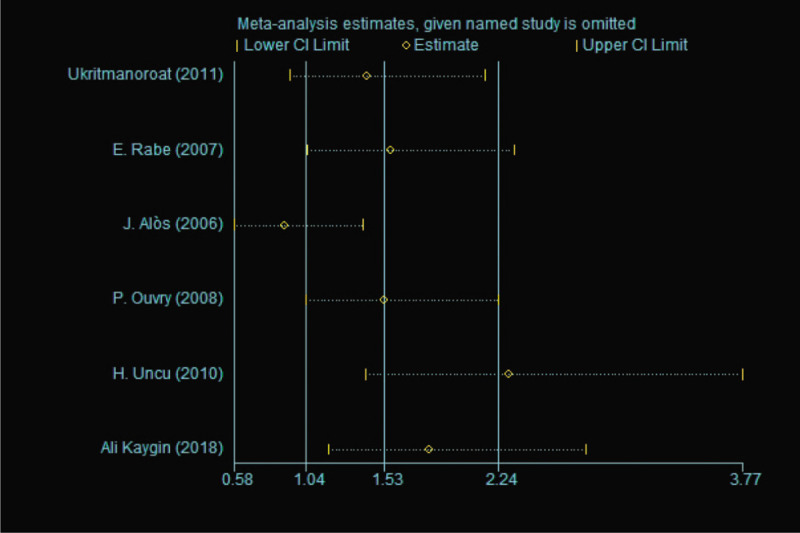
Sensitivity analysis of Incidence rate of hyperpigmentation between foam sclerotherapy and liquid sclerotherapy.

**Figure 10:**
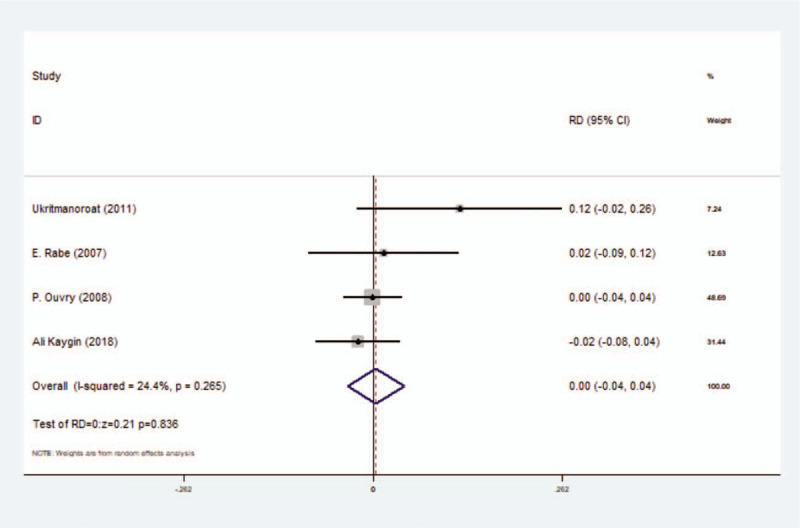
Forest plot to assess Incidence rate of hyperpigmentation between foam sclerotherapy and liquid sclerotherapy after exclusion.

Egger test was performed to assess the publication bias in this meta-analysis, with *P* = .911 (*P* > .05) (data were not shown).^[14]^ Incidence rate of local inflammation showed a high heterogeneity (*I*^2^ = 64.9%) and we performed the sensitivity analysis to explore its reasons. From Figure 7, we found that the third study (J. Alòs 2006)^[17]^ and the last study (Ali Kaygin 2018)^[20]^ had great impacts on statistic and we found that the heterogeneity reduced dramatically after exclusion of these two studies (Fig. 8). As for incidence rate of hyperpigmentation, we used the same analysis and we found that J. Alòs 2006 and H. Uncu 2010 maybe reasons of its high hetergeneity (Figs. 9 and 10).

## 4 Discussion

Lower extremity varicose veins is a commonly chronic medical condition and a substantial cause of morbidity around the world.^[23]^ The natural history of this kind of diseases is progressive over time, ranging from simple telangiectasias to venous ulcerations if treated inappropriately, leading to a huge amount of care and costs.^[24,25]^ Treatments of lower extremity varicose veins are diversified and could be divided into two parts: surgical strategy (e.g., high ligation and stripping, stab avulsion phlebectomy, valvuloplasty) and non-surgical strategy (RFA, EVLA, compression therapy).

As one of the emerging and promising treatments, sclerotherapy with foam sclerosant or liquid sclerosant has enjoyed a great popularity nowadays. Sclerosants could destroy vascular cell membranes, leading to continued vasospasm and denudation of the venous monolayer.^[26]^ A series of pathological changes would cause irreversible fibrous obliteration of lesional veins, resulting in occluding varicose veins for therapeutic purposes.^[27]^ However, with the invention and development of foam sclerosant, hot debate on the clinical efficacy for lower extremity varicose veins of foam sclerotherapy versus liquid sclerotherapy. To date, this is the first meta-analysis to compare these two different sclerosants and offer clinical practitioners and stakeholders sufficient scientific evidence for the clinical efficacy of foam sclerotherapy as an optimal treatment for patients suffering lower extremity varicose veins.

## 5 Summary of evidence

Our study manifests that although foam sclerotherapy might make some patients more painful than liquid sclerotherapy, foam group have a significantly higher effective rate than liquid group. Furthermore, as for other complications analyzed by us, such as local inflammation, thrombophlebitis, hyperpigmentation, there were no significant differences among them between two groups. Furthermore, Egger test showed that there was no significant publication bias in this meta-analysis. Our results reflect an authentic clinical efficacy of foam sclerotherapy and liquid sclerotherapy, rather than an artefact of statistical heterogeneity.

Yamaki et al^[28]^ and Gibson et al^[29]^ reported that foam sclerotherapy was better than liquid sclerotherapy because foam sclerosant could contact with venous endothelial for a longer period than would occur with the injection of liquid. A RCT published by Hamel-Desnos C et al^[21]^ also showed that closure rates of the great saphenous vein (GSV) treated with foam sclerotherapy were higher than that of liquid sclerotherapy (3 weeks: 84% vs 40%; 6 weeks: 80% vs 26%). Nevertheless, Kathleen Gibson et al^[30]^ reported that liquid sclerotherapy was preferred for spider telangiectasias, but when it came to truncal veins such as lower extremity varicose veins, foam sclerotherapy were superior. Although there were relative studies giving us similar conclusions, the level of each evidences was low and the amount of patients of each study was small. But our meta-analysis was the first high-level clinical evidence of which the sample size was large (>1000 patients), so our conclusions would be more convinced and could offer clinical pratitioners valuable practice guideline.

To make this study more scientific and rational, after a thorough screen of included studies, we extracted some important data based on such six clinical and objective evaluation indicators such length of the sclerosed veins, reduced mean influx time, required sclerotherapy sessions, recanalization rate, Venous Clinical Severity Scoring (VCSS) and complete disappearance rate of varicose veins (Table 4). From outcomes we could include that compared with liquid sclerotherapy, foam sclerotherapy could achieve better treatment effects when it comes to length of the sclerosed veins, reduced mean influx time, required sclerotherapy sessions, Venous Clinical Severity Scoring (VCSS) and complete disappearance rate of varicose veins. In addition, the recurrence rate of foam sclerotherapy is lower than liquid sclerotherapy (recanalization rate, 4.55% vs 13.64%).

**Table 4:**
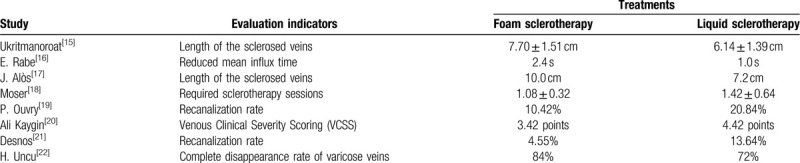
Evaluation indicators of treatments for lower extremity varicose veins.

## 6 Limitations

However, there were some limitations in this meta-analysis. First, all included studies were from Asia and Europe, so included participants were Asian ethnicity and European ethnicity in this meta-analysis. Therefore, our study may be not applicable to African ethnicity.

Secondly, only 5 outcomes were evaluated and analyzed in our study, which would deteriorate the persuasion and rationality of our conclusions inevitably. Moreover, there were other complications after sclerotherapy such as immediate spasm, hematoma, itching, hypertension et al. We did not collect these complications because its incidence rates were rare and relative studies were not enough to be analyzed.

Thirdly, the evaluation methods of each study were not uniform and the majority of studies (5/8) mainly depended on clinical experience. It would bring about some biases during the trials because clinical experience was very subjective and it would vary easily with different evaluators, hospitals, and countries.

## 7 Conclusions

In summary, although foam sclerotherapy has a higher incidence rate of complications, it could achieve a more stable clinical efficacy in the treatment of lower extremity varicose veins than liquid sclerotherapy. Moreover, based on the above conclusions we had draw, more high quality researches should be performed to determine how to optimize foam sclerosant in order to reduce the incidence rate of complications after injections.

## Author contributions

**Data curation:** minglei Bi.

**Formal analysis:** minglei Bi.

**Methodology:** danyi Li.

**Software:** minglei Bi, danyi Li, weina Zhang.

**Supervision:** weina Zhang.

**Writing – original draft:** zhenyu Chen, weina Zhang.

**Writing – review & editing:** zhenyu Chen, yanjin Wang, jizhen Ren, weina Zhang.
